# The Analgesic Efficacy of Adding Dexmedetomidine to Ropivacaine in Preoperative Interscalene Block for Arthroscopic Rotator Cuff Repair

**DOI:** 10.5812/aapm-134057

**Published:** 2026-06-30

**Authors:** Mahmood-Reza Alebouyeh, Saeed Reza Entezary, Shirin Zarandooz, Masood Mohseni

**Affiliations:** 1 Department of Anesthesiology and Pain, Medical of School, Iran University Medical Science (I. U. M. S) Anesthesiologist and Pain Fellowship Tehran Iran

**Keywords:** Ropivacaine, Dexmedetomidine, Arthroscopic Shoulder Surgery, Interscalene Block, Rotator Cuff, Regional Anesthesia

## Abstract

**Background:**

Effective postoperative pain management is essential after arthroscopic shoulder surgery. Ultrasound-guided interscalene block (ISB) is widely used for perioperative analgesia; however, the benefits of adding dexmedetomidine to local anesthetics remain uncertain.

**Objectives:**

This study aimed to evaluate the analgesic efficacy and hemodynamic effects of dexmedetomidine as an adjuvant to ropivacaine for ultrasound-guided ISB in patients undergoing arthroscopic rotator cuff repair.

**Methods:**

This double-blind randomized clinical trial included patients aged ≥ 18 years with American Society of Anesthesiologists physical status I-III who were scheduled for elective arthroscopic rotator cuff repair. Forty-two patients were randomly allocated in a 1:1 ratio using a random-number table to receive ultrasound-guided ISB with either 20 mL of 0.25% ropivacaine plus 2 mL of normal saline (R group) or 20 mL of 0.25% ropivacaine plus dexmedetomidine (1.5 μg/kg), diluted to a total volume of 2 mL (ropivacaine–dexmedetomidine (RD) group). Randomization and preparation of the study solutions were performed by an independent investigator. The anesthesiologist performing the block, the patients, and the postoperative outcome assessor remained blinded to the treatment allocation. Pain intensity was assessed using the Numerical Rating Scale (NRS) in the recovery room and at 2, 6, 18, and 24 hours postoperatively. Intraoperative remifentanil consumption, time to the first patient-controlled intravenous analgesia (PCIA) request, number of PCIA requests, pethidine requirements, and hemodynamic variables were recorded. Data were analyzed using the Kolmogorov–Smirnov test, independent-samples t-test, Mann–Whitney U test, chi-square test, or Fisher’s exact test, as appropriate.

**Results:**

Forty patients completed the study and were included in the per-protocol analysis (20 patients in each group). Compared with the R group, the RD group had significantly lower intraoperative remifentanil consumption (0.11 ± 0.02 vs 0.24 ± 0.04 μg/kg/min, P < 0.001), a longer time to the first PCIA request (17.57 ± 0.93 vs 13.37 ± 1.25 hours after ISB, P < 0.001), fewer PCIA requests (4.50 ± 1.19 vs 9.95 ± 1.50, P < 0.001), a longer time to the first pethidine administration (19.20 ± 0.83 vs 16.33 ± 1.52 hours after ISB, P < 0.001), and fewer pethidine administrations (0.35 ± 0.67 vs 3.45 ± 1.05, P < 0.001). NRS pain scores at 18 and 24 hours were also significantly lower in the RD group. No significant between-group differences were observed in systolic blood pressure, diastolic blood pressure, and heart rate.

**Conclusions:**

The addition of dexmedetomidine to ropivacaine for an ultrasound-guided interscalene block significantly prolonged postoperative analgesia, reduced intraoperative remifentanil consumption, decreased postoperative analgesic requirements, and improved postoperative pain control, without significant adverse effects on hemodynamic parameters, in patients undergoing arthroscopic rotator cuff repair.

## 1. Background

Postoperative pain after arthroscopic shoulder surgery remains a major clinical concern because it can delay rehabilitation, prolong hospital stay, and reduce patient satisfaction. Effective pain control facilitates early mobilization, improves functional recovery, and decreases opioid consumption, making postoperative analgesia an essential component of perioperative care (1, 2).

Ultrasound-guided interscalene block (ISB) is considered the regional anesthesia technique of choice for shoulder surgery because it provides excellent perioperative analgesia, reduces opioid requirements, and improves postoperative recovery. However, the duration of analgesia achieved with local anesthetics alone is limited, prompting investigation of adjuvant agents that may prolong block duration and enhance analgesic efficacy (3).

Dexmedetomidine is a highly selective α2-adrenergic receptor agonist with sedative, analgesic, anxiolytic, and sympatholytic properties. As an adjuvant to peripheral nerve blocks, dexmedetomidine has been reported to prolong sensory and motor block duration, enhance postoperative analgesia, reduce opioid consumption, and improve patient comfort. These effects are thought to result from both peripheral and central mechanisms, including inhibition of norepinephrine release, hyperpolarization of nerve membranes, and modulation of nociceptive transmission. Nevertheless, dexmedetomidine may also produce dose-dependent adverse effects, such as bradycardia and hypotension, necessitating careful evaluation of its safety profile (4-7).

Several clinical studies have investigated dexmedetomidine as an adjunct to local anesthetics for peripheral nerve blocks; however, the magnitude of its analgesic benefit and its effects on perioperative hemodynamic stability remain inconsistent across studies. In particular, evidence regarding its use with ropivacaine for ultrasound-guided ISB in patients undergoing arthroscopic rotator cuff repair remains limited (8, 9).

## 2. Objectives

Therefore, this double-blind randomized clinical trial aimed to determine whether the addition of dexmedetomidine to ropivacaine for ultrasound-guided interscalene block improves postoperative analgesia and reduces perioperative analgesic requirements without adversely affecting hemodynamic stability in patients undergoing arthroscopic rotator cuff repair.

## 3. Methods

### 3.1. Study Design and Participants

This double-blind randomized clinical trial included adult patients undergoing elective arthroscopic rotator cuff repair who were scheduled to receive an ultrasound-guided ISB. Eligible patients were aged ≥ 18 years and had American Society of Anesthesiologists physical status I-III.

Patients were excluded if they were pregnant; had a history of asthma, chronic obstructive pulmonary disease, diabetes mellitus, coagulopathy, cardiac conduction abnormalities (e.g., heart block), head trauma, or forearm deformity; had received opioids within 3 - 5 days before surgery; or had any contraindication to an interscalene block, including diaphragmatic paralysis.

### 3.2. Randomization and Blinding

Participants were randomly assigned in a 1:1 ratio using a random-number table. Randomization, group allocation, and preparation of the study solutions were performed by an independent investigator who was not involved in block performance, intraoperative management, postoperative assessment, or data analysis. The study solutions were prepared in identical syringes with the same total volume to ensure allocation concealment.

Participants, the orthopedic surgeon, the postoperative outcome assessor, and investigators responsible for follow-up remained blinded to treatment allocation throughout the study. The anesthesiologist performing the interscalene block was not involved in randomization, postoperative outcome assessment, or data analysis.

### 3.3. Study Interventions

After admission to the preoperative area, standard monitoring, including electrocardiography, noninvasive blood pressure measurement, and pulse oximetry, was established. A peripheral intravenous catheter was inserted, and all patients received 500 mL of Ringer’s solution before the block.

Patients in the ropivacaine (R) group received an ultrasound-guided interscalene block with 20 mL of 0.25% ropivacaine plus 2 mL of normal saline. Patients in the ropivacaine–dexmedetomidine (RD) group received 20 mL of 0.25% ropivacaine combined with dexmedetomidine (1.5 μg/kg) diluted to a total volume of 2 mL (9).

The ultrasound-guided interscalene block was performed using the classical approach. After the brachial plexus was identified between the anterior and middle scalene muscles, the study solution was injected under ultrasound guidance. Block success was confirmed by pinprick sensory testing and assessment of analgesia during passive shoulder movement before transfer to the operating room.

All patients received standardized general anesthesia. Premedication consisted of midazolam (1 mg), lidocaine (1 mg/kg), and fentanyl (3 μg/kg). General anesthesia was induced with propofol (2 mg/kg) and atracurium (0.5 mg/kg), followed by endotracheal intubation. Anesthesia was maintained with propofol using bispectral index (BIS) monitoring (target bispectral index 40 - 60), remifentanil infusion, and supplemental atracurium guided by train-of-four monitoring. Intravenous fluids were administered according to body weight and estimated blood loss. Hypotension, defined as a decrease in systolic blood pressure greater than 30% from baseline, was treated with ephedrine (10 mg), whereas bradycardia (heart rate < 45 beats/min) was treated with atropine (0.5 mg).

### 3.4. Outcome Assessment

Hemodynamic variables, including systolic blood pressure, diastolic blood pressure, and heart rate, were recorded immediately before the interscalene block and 1 hour thereafter.

After surgery, patients were transferred to the recovery room. Once patients had fully recovered from anesthesia, postoperative pain was assessed using the Numerical Rating Scale (NRS), and sedation was evaluated using the Ramsay Sedation Scale. Pain assessments were repeated at 2, 6, 18, and 24 hours after surgery by a blinded investigator.

Postoperative analgesia was provided using patient-controlled intravenous analgesia (PCIA) containing Apotel (1 g), fentanyl (5 mL), and normal saline to a total volume of 50 mL. The times to the first PCIA request and the first pethidine administration were measured from ISB. These intervals and the total number of PCIA requests were recorded during the first 24 postoperative hours. Intravenous pethidine (20 mg) was administered when the NRS score exceeded 4.

Adverse events related to the interscalene block, including nausea, vomiting, pneumothorax, hematoma, neurological complications, local anesthetic systemic toxicity, hypotension, and bradycardia, were recorded throughout the study.

Patients were followed up at 3 and 6 months after surgery through outpatient visits or video consultations. Persistent shoulder pain and limitation of active and passive shoulder motion were assessed during follow-up.

Two randomized participants were excluded from the per-protocol analysis after their surgeries were canceled because of adverse events following the interscalene block. One participant developed pneumothorax, whereas another experienced hemodynamic instability before surgery.

### 3.5. Statistical Analysis

Continuous variables are presented as mean ± standard deviation (SD), whereas categorical variables are presented as frequencies and percentages. Normality was assessed using the Kolmogorov–Smirnov test. Continuous variables were compared using the independent-samples t-test or the Mann–Whitney U test, as appropriate. Categorical variables were analyzed using the chi-square test or Fisher’s exact test when expected cell counts were less than five. Statistical analyses were performed using SPSS software version 23.0 (IBM Corp., Armonk, NY, USA). The primary analysis followed the per-protocol principle. A two-sided P value < 0.05 was considered statistically significant.

## 4. Results

A total of 42 patients were randomized. One participant in the ropivacaine–dexmedetomidine (RD) group developed pneumothorax following an interscalene block, and one participant in the ropivacaine (R) group experienced hemodynamic instability before surgery. In both cases, the scheduled surgery was canceled; therefore, these two participants were excluded from the per-protocol analysis. Consequently, data from 40 patients (20 in each group) were included in the final analysis (Figure 1).

**Figure 1.**
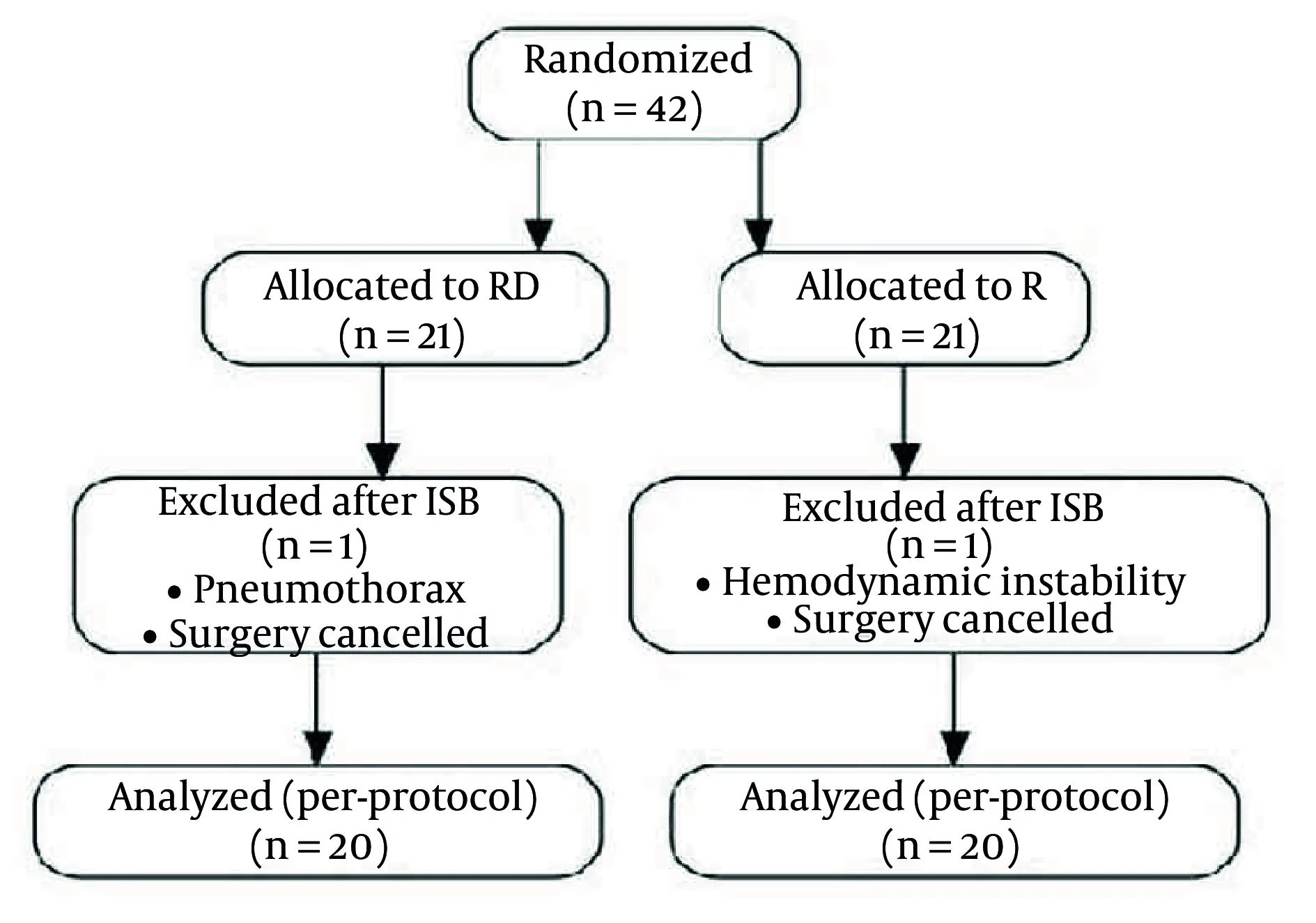
Total analyzed (per-protocol): n = 40

Baseline demographic characteristics were comparable between the two groups. No significant differences were observed in sex distribution (80% vs 70% male, P = 0.465), mean age (36.45 ± 10.89 vs 36.10 ± 11.52 years, P = 0.922), or body weight (78.65 ± 7.76 vs 79.90 ± 8.99 kg, P = 0.641) between the RD and R groups, respectively (Table 1).

**Table 1.** Comparison of Sex, Age, and Weight Distribution ^a^

Variables	RD (n = 20)	R (n = 20)	P Value
**Gender**			0.465
Male	80	70	
Female	20	30	
**Age (y)**	36.45 ± 10.89	36.10 ± 11.52	0.922
**Weight (kg)**	78.65 ± 7.76	79.90 ± 8.99	0.641

^a^ Values are expressed as percentage or mean ± SD. Abbreviations: RD, ropivacaine–dexmedetomidine; R, ropivacaine.

Regarding intraoperative anesthetic requirements, no significant difference was observed in the mean propofol infusion rate between the RD and R groups (88.15 ± 4.54 vs 89.95 ± 4.22 μg/kg/min, P = 0.202). Likewise, atracurium requirements were comparable between the two groups (P = 0.650). However, patients in the RD group required significantly less remifentanil than those in the R group (0.11 ± 0.02 vs 0.24 ± 0.04 μg/kg/min, P < 0.001) (Figure 2).

**Figure 2.**
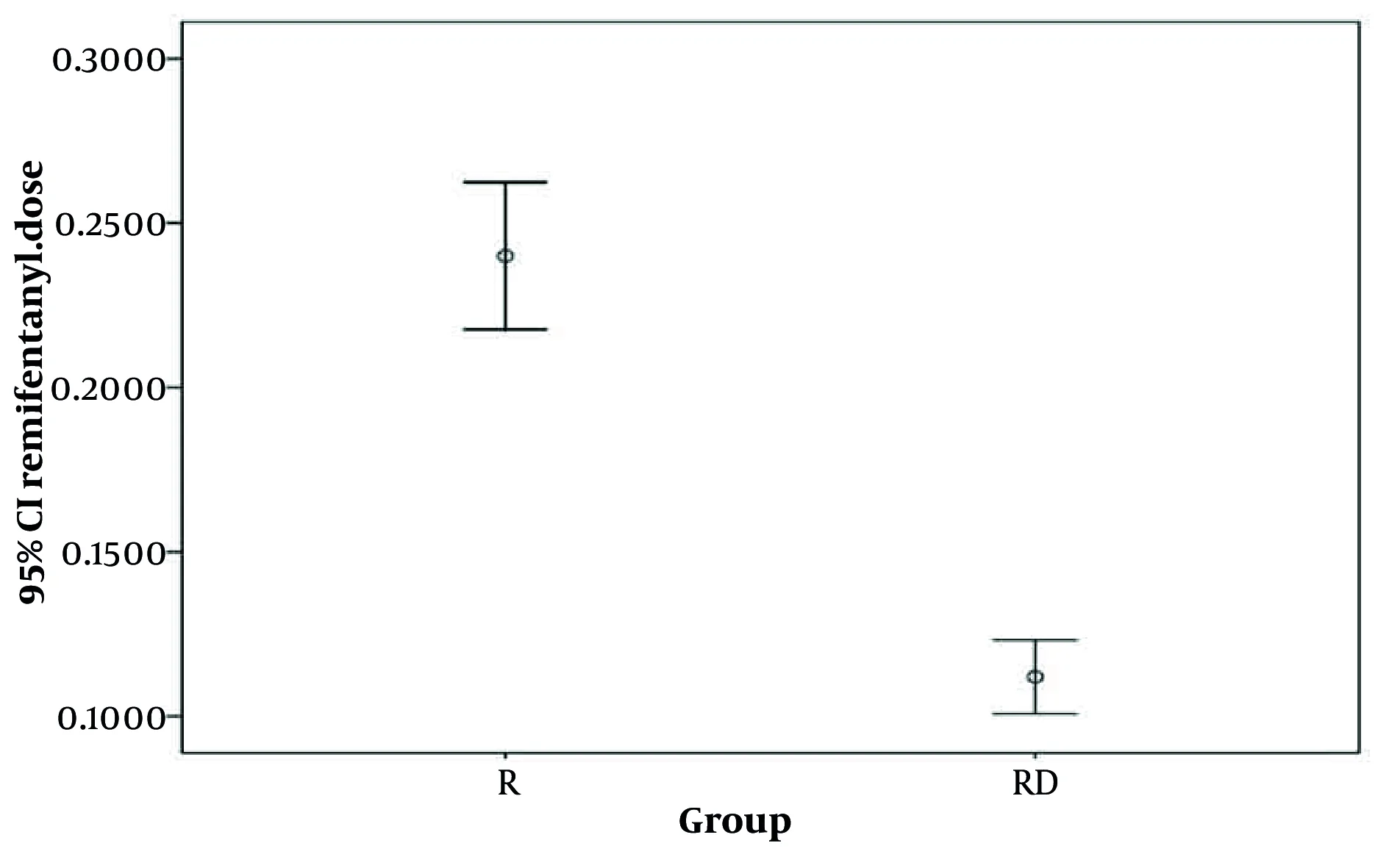
Remifentanil dosage,microgram/kg/min

The mean time to the first PCIA request was significantly longer in the RD group than in the R group (17.57 ± 0.93 vs 13.37 ± 1.25 hours after ISB, P < 0.001) (Figure 3). In addition, the RD group made significantly fewer PCIA requests during the first 24 postoperative hours than the R group (4.50 ± 1.19 vs 9.95 ± 1.50, P < 0.001) (Figure 4).

**Figure 3.**
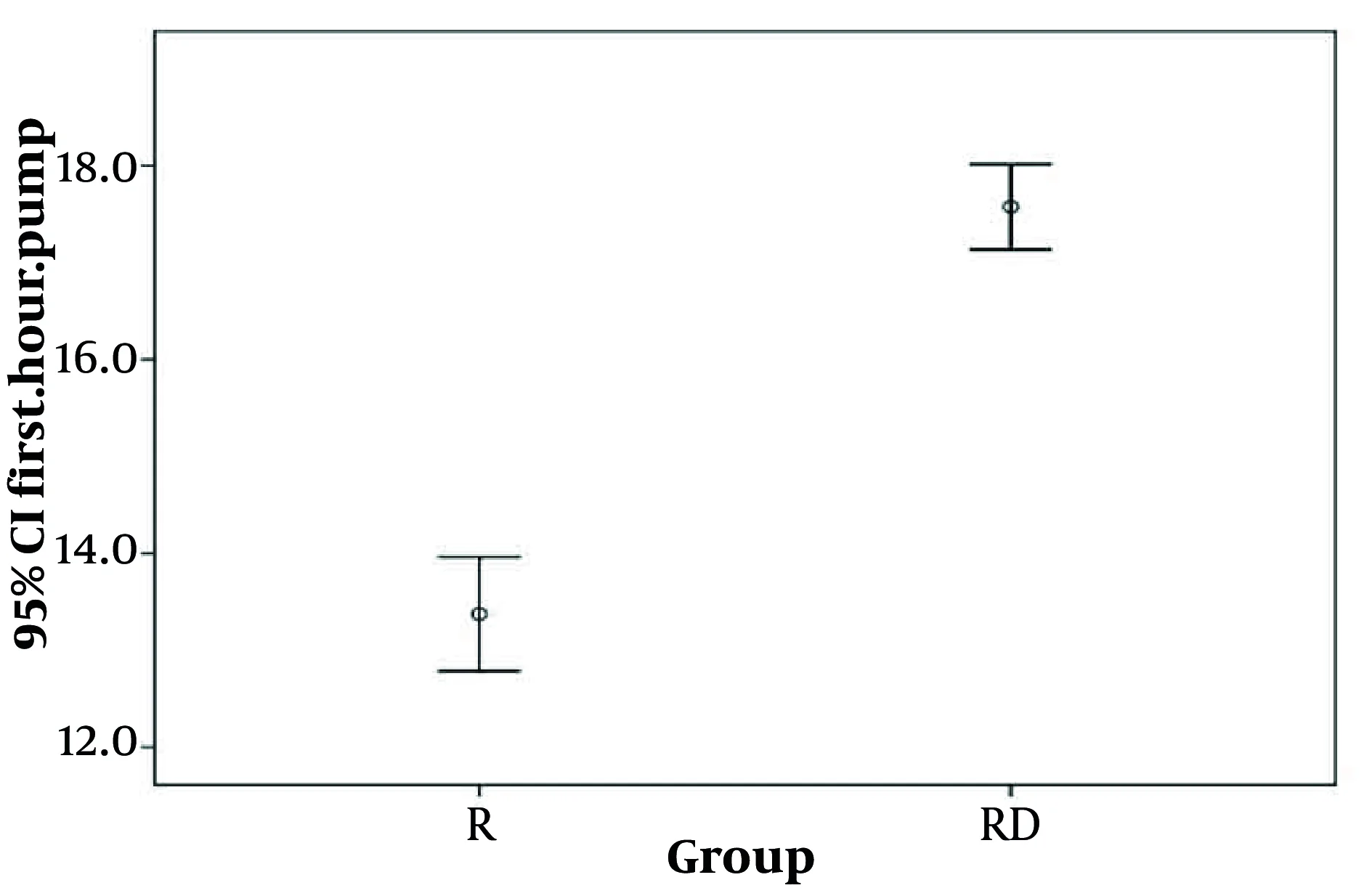
Mean time to first patient-controlled intravenous analgesia (PCIA) Pump request

**Figure 4.**
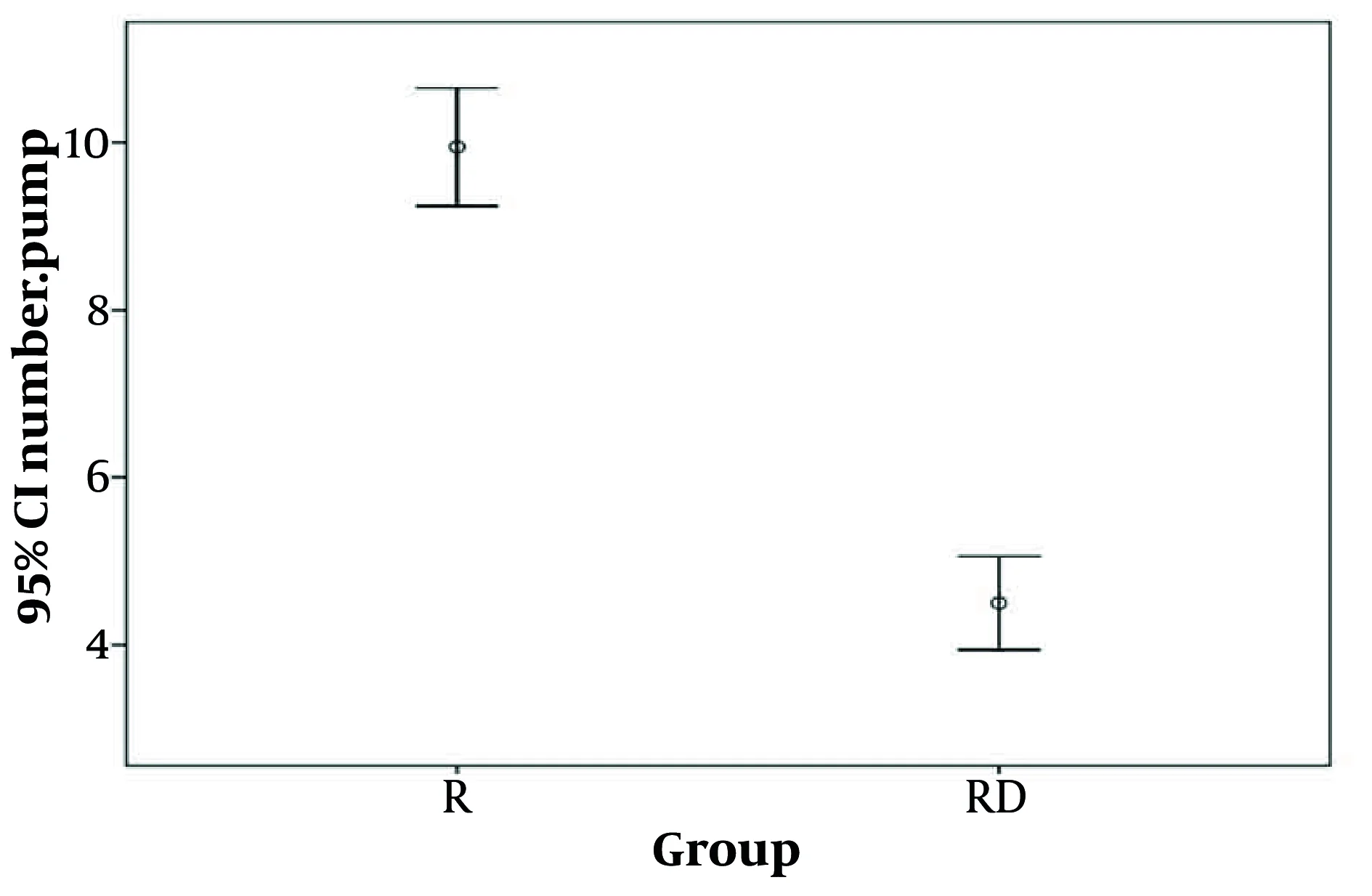
Mean Number of patient-controlled intravenous analgesia (PCIA) Pump Requests

Similarly, the time to the first administration of rescue pethidine was significantly longer in the RD group than in the R group (19.20 ± 0.83 vs 16.33 ± 1.52 hours after ISB, P < 0.001) (Figure 5). Furthermore, patients in the RD group required significantly fewer pethidine administrations during the first 24 postoperative hours than those in the R group (0.35 ± 0.67 vs 3.45 ± 1.05 administrations, P < 0.001) (Figure 6).

**Figure 5.**
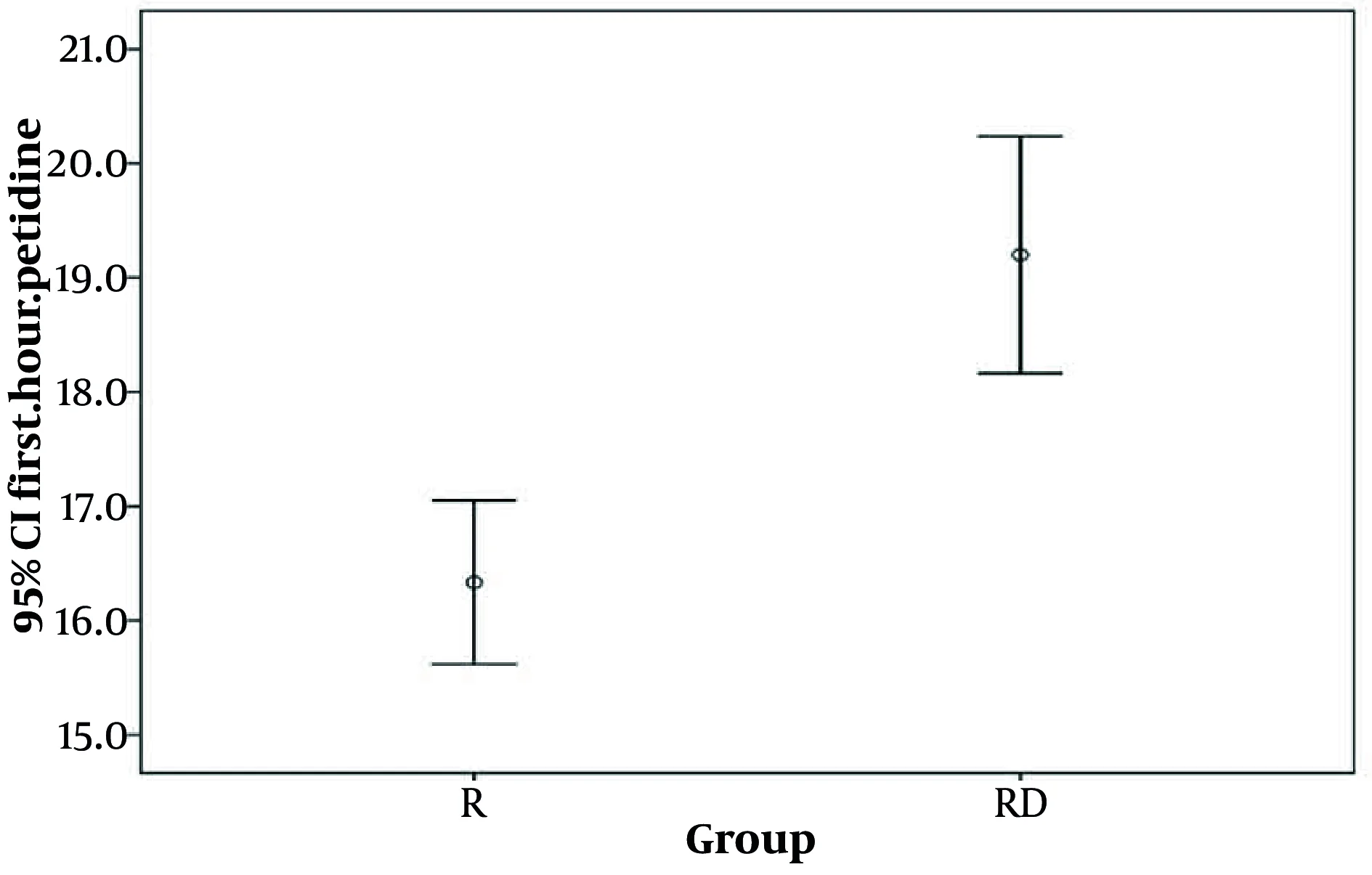
Mean time to first pethidine administration

**Figure 6.**
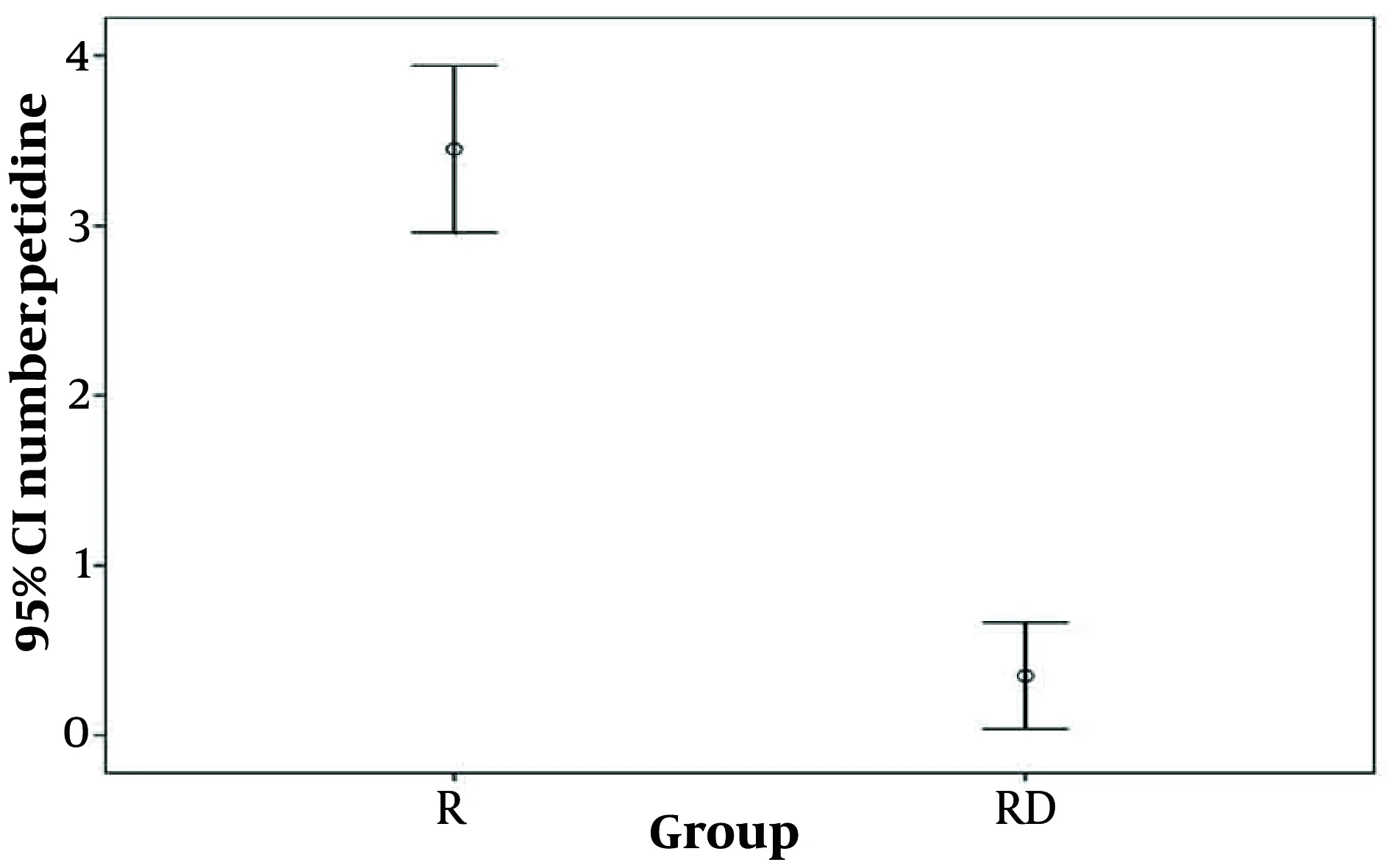
Mean Times of pethidine administration

Postoperative pain scores are presented in Table 2. Numerical Rating Scale scores at 2 and 6 hours after surgery were zero in both groups. However, at 18 and 24 hours, pain scores were significantly lower in the RD group than in the R group (both P < 0.001).

**Table 2.** Comparison of Numerical Rating Scale Scores ^a^

Variables	RD	R	P Value
**Pain score (NRS), h**			0.001
2	0	0	
6	0	0	
18	2.35 ± 0.81	4.50 ± 0.52	
24	1.70 ± 0.57	3.05 ± 0.68	

^a^ Abbreviations: RD, ropivacaine–dexmedetomidine; R, ropivacaine; NRS, Numerical Rating Scale. Times are measured after surgery.

No statistically significant differences were observed between the two groups in systolic blood pressure, diastolic blood pressure, or heart rate either before ISB or 1 hour after the block (Table 3).

**Table 3.** Comparison of Hemodynamic Status ^a^

Variables	RD (n = 20)	R (n = 20)	P Value
**Mean systolic blood pressure (mm Hg)**			
Baseline	122.10 ± 9.44	123.05 ± 9.16	0.950
1 h later	110.70 ± 7.94	111.50 ± 7.11	0.800
**Mean diastolic blood pressure (mm Hg)**			
Baseline	65.15 ± 4.56	65.70 ± 5.74	0.739
1 h later	61.80 ± 3.84	64.35 ± 4.53	0.083
**Mean heart rate (beats/min)**			
Baseline	81.60 ± 7.38	80.85 ± 6.65	0.738
1 h later	68.75 ± 5.18	72.95 ± 5.38	0.086

^a^ Abbreviations: RD, ropivacaine–dexmedetomidine; R, ropivacaine. Baseline measurements were obtained immediately before interscalene block.

At the 3- and 6-month follow-up assessments, no significant differences were observed between the two groups in persistent shoulder pain or limitations in active and passive shoulder movements. Similarly, the incidence of postoperative shoulder stiffness was comparable between the RD and R groups (P = 0.605).

## 5. Discussion

The present double-blind randomized clinical trial demonstrated that adding dexmedetomidine (1.5 μg/kg) to ropivacaine for ultrasound-guided interscalene block significantly improved postoperative analgesia in patients undergoing arthroscopic rotator cuff repair. Compared with ropivacaine alone, dexmedetomidine prolonged the interval from ISB to the first postoperative analgesic request, reduced intraoperative remifentanil consumption, decreased postoperative opioid requirements, and resulted in lower pain scores at 18 and 24 hours after surgery. No statistically significant differences were observed between the two groups in systolic blood pressure, diastolic blood pressure, or heart rate.

The analgesic benefits observed in the present study are biologically plausible and consistent with the pharmacological profile of dexmedetomidine. As a highly selective α2-adrenergic receptor agonist, dexmedetomidine enhances the effects of local anesthetics through peripheral and central mechanisms, including inhibition of norepinephrine release, hyperpolarization of nerve membranes, and modulation of nociceptive transmission (7). These mechanisms may contribute to prolonged postoperative analgesia and reduced perioperative opioid consumption (7). In the present study, a dose of 1.5 μg/kg was selected based on previous clinical evidence demonstrating an acceptable balance between analgesic efficacy and safety; higher doses have been associated with increased risks of bradycardia and hypotension (9).

Our findings are consistent with those reported by Fritsch et al., who demonstrated that adding dexmedetomidine significantly prolonged the duration of the interscalene block and reduced postoperative pain intensity compared with a local anesthetic alone (8). Although Fritsch et al. observed a reduction in heart rate in the dexmedetomidine group, we did not detect significant differences in heart rate or blood pressure between the study groups. This discrepancy may be attributable to differences in dexmedetomidine dose, patient characteristics, anesthetic management, or perioperative monitoring protocols.

Similarly, Rashmi et al. reported that dexmedetomidine accelerated the onset of sensory and motor block while significantly prolonging block duration when combined with ropivacaine (10). Dai et al. also demonstrated that dexmedetomidine shortened the onset time of both sensory and motor block and prolonged postoperative analgesia without increasing the incidence of clinically significant hypotension (11). Although block onset and duration were not directly measured in the present study, the prolonged time to the first postoperative analgesic request and the reduced postoperative opioid requirements observed in our patients support the enhanced analgesic efficacy of dexmedetomidine as an adjuvant to ropivacaine.

Kataria et al. likewise reported a significantly prolonged duration of postoperative analgesia and lower postoperative fentanyl consumption in patients receiving dexmedetomidine as an adjuvant to ropivacaine. In addition, patient satisfaction was higher, without an increase in postoperative adverse events (12). These findings agree with our results, which demonstrated reduced intraoperative remifentanil consumption, lower postoperative pethidine requirements, and improved postoperative pain control in the dexmedetomidine group.

Other publications provide context for postoperative pain management but address different populations, interventions, or anatomical sites. Divizia et al. described suprascapular nerve neuromodulation for chronic shoulder pain (13). Ismail et al. and Imani et al. investigated dexmedetomidine as an adjuvant in pediatric caudal anesthesia rather than interscalene block (14, 15). Imani et al. examined dexmedetomidine for the treatment of radicular low-back pain (16). Pehora et al. reviewed dexamethasone, a different peripheral nerve block adjuvant (17). Omar Mostafa et al. evaluated dexmedetomidine in paravertebral block for mastectomy (18), whereas Imani et al. investigated magnesium sulfate added to ropivacaine for transversus abdominis plane block (19). Given these differences, these reports cannot be considered direct evidence for the efficacy or safety of dexmedetomidine in interscalene block.

In contrast, Margulis et al. found that although dexmedetomidine reduced intraoperative opioid requirements, it did not significantly decrease postoperative morphine consumption or improve postoperative pain scores compared with ropivacaine alone (20). Differences in study design, patient populations, dexmedetomidine dosage, local anesthetic concentration, surgical procedures, outcome measures, and peripheral nerve block techniques may partly explain the discrepancy between their findings and those of the present study. Dexmedetomidine has also been compared with remifentanil in posterior spinal fusion surgery, although that setting is not directly comparable with the use of an interscalene block (21).

### 5.1. Conclusions

In conclusion, adding dexmedetomidine to ropivacaine for ultrasound-guided interscalene block in patients undergoing arthroscopic rotator cuff repair was associated with improved postoperative analgesia, reduced intraoperative remifentanil consumption, decreased postoperative pethidine requirements, and lower postoperative pain scores compared with ropivacaine alone. No statistically significant differences were observed between the two groups in hemodynamic parameters. Further large-scale, multicenter randomized trials are needed to confirm these findings and to determine the optimal dose of dexmedetomidine as an adjuvant to peripheral nerve blocks.

## Data Availability

The dataset presented in the study is available on request from the corresponding author during submission or after publication.

## Clinical Trial Registration Code:

IRCT20181011041308N4.

## References

[1] Peters CL, Shirley B, Erickson J (2006). The effect of a new multimodal perioperative anesthetic regimen on postoperative pain, side effects, rehabilitation, and length of hospital stay after total joint arthroplasty. J Arthroplasty.

[2] Malik KM, Imani F, Beckerly R, Chovatiya R (2020). Risk of opioid use disorder from exposure to opioids in the perioperative period: A systematic review. Anesth Pain Med.

[3] Faiz SHR, Mohseni M, Imani F, Attaee MK, Movassaghi S, Rahimzadeh P (2021). Comparison of ultrasound-guided supra-scapular plus axillary nerve block with interscalene block for postoperative pain management in arthroscopic shoulder surgery; a double-blinded randomized open-label clinical trial. Anesth Pain Med.

[4] Imani F, Zaman B, De Negri P (2020). Postoperative pain management: Role of dexmedetomidine as an adjuvant. Anesth Pain Med.

[5] Marhofer D, Kettner SC, Marhofer P, Pils S, Weber M, Zeitlinger M (2013). Dexmedetomidine as an adjuvant to ropivacaine prolongs peripheral nerve block: A volunteer study. Br J Anaesth.

[6] Edinoff AN, Houk GM, Patil S, Bangalore Siddaiah H, Kaye AJ, Iyengar PS (2021). Adjuvant Drugs for Peripheral Nerve Blocks: The Role of Alpha-2 Agonists, Dexamethasone, Midazolam, and Non-Steroidal Anti-Inflammatory Drugs. Anesth Pain Med.

[7] Vorobeichik L, Brull R, Abdallah FW (2017). Evidence basis for using perineural dexmedetomidine to enhance the quality of brachial plexus nerve blocks: A systematic review and meta-analysis of randomized controlled trials. Br J Anaesth.

[8] Fritsch G, Danninger T, Allerberger K, Tsodikov A, Felder TK, Kapeller M (2014). Dexmedetomidine added to ropivacaine extends the duration of interscalene brachial plexus blocks for elective shoulder surgery when compared with ropivacaine alone: A single-center, prospective, triple-blind, randomized controlled trial. Reg Anesth Pain Med.

[9] Jung HS, Seo KH, Kang JH, Jeong JY, Kim YS, Han NR (2018). Optimal dose of perineural dexmedetomidine for interscalene brachial plexus block to control postoperative pain in patients undergoing arthroscopic shoulder surgery. Medicine (Baltimore).

[10] Rashmi HD, Komala HK (2017). Effect of Dexmedetomidine as an Adjuvant to 0.75% Ropivacaine in Interscalene Brachial Plexus Block Using Nerve Stimulator: A Prospective, Randomized Double-blind Study. Anesth Essays Res. Jan-Mar.

[11] Dai W, Tang M, He K (2018). The effect and safety of dexmedetomidine added to ropivacaine in brachial plexus block: A meta-analysis of randomized controlled trials. Medicine (Baltimore).

[12] Kataria S, Mitra S, Saroa R, Jindal S, Gupta R (2019). Randomized Double Blinded Trial Comparing Dexmedetomidine With Dexamethasone as an Adjunct to Ropivacaine in Ultrasound Guided Interscalene Block for Arthroscopic Shoulder Surgery. Asian J Anesthesiol.

[13] Divizia M, Germani G, Urti I, Imani F, Varrassi G, Meloncelli S (2020). Endoscopic neuromodulation of suprascapular nerve in chronic shoulder pain: A case report. Anesth Pain Med.

[14] Ismail AA, Mohamed Hamza H, Ali Gado A (2021). Efficacy of dexmedetomidine versus morphine as an adjunct to bupivacaine in caudal anesthesia for pediatric thoracic surgeries: A randomized controlled trial. Anesth Pain Med.

[15] Imani F, Farahmand Rad R, Salehi R, Alimian M, Mirbolook Jalali Z, Mansouri A (2021). Evaluation of adding dexmedetomidine to ropivacaine in pediatric caudal epidural block: A randomized, double-blinded clinical trial. Anesth Pain Med.

[16] Imani F, Rahimzadeh P, Khademi H, Narimani Zamanabadi M, Sadegi K, Abolfazli A (2019). Comparison of transforaminal triamcinolone and dexmedetomidine in radicular low-back pain: A randomized double-blind clinical trial. Anesth Pain Med.

[17] Pehora C, Pearson AM, Kaushal A, Crawford MW, Johnston B (2017). Dexamethasone as an adjuvant to peripheral nerve block. Cochrane Database Syst Rev.

[18] Omar Mostafa M, Makram Botros J, Sayed Khaleel AM (2018). Effect of dexmedetomidine versus nalbuphine as an adjuvant on paravertebral block to manage postoperative pain after mastectomies. Anesth Pain Med.

[19] Imani F, Rahimzadeh P, Faiz HR, Baghaei AR (2018). An evaluation of the adding magnesium sulfate to ropivacaine on ultrasound-guided transverse abdominis plane block after abdominal hysterectomy. Anesth Pain Med.

[20] Margulis R, Francis J, Tischenkel B, Bromberg A, Pedulla D, Grtisenko K (2021). Comparison of dexmedetomidine and dexamethasone as adjuvants to ultra-sound guided interscalene block in arthroscopic shoulder surgery: A double-blinded randomized placebo-controlled study. Anesth Pain Med.

[21] Rahimzadeh P, Faiz SH, Alimian M, Mohammadian Erdi A (2015). Remifentanil versus dexmedetomidine for posterior spinal fusion surgery. Med J Islam Repub Iran.

